# P25 Polymicrobial UTI: could Lodestar DX be used to differentiate between contamination or true infection?

**DOI:** 10.1093/jacamr/dlae217.029

**Published:** 2025-01-23

**Authors:** Joanna Diggle, Stuart Drazich-Taylor, James Moore, Brian McCann, John Hayward, Emma Hayhurst

**Affiliations:** Llusern Scientific, Cardiff Edge Business Park, Cardiff, UK; Norwich and Norfolk University Hospital, Colney Ln, Norwich, UK; University of East Anglia, Research Park, Norwich, UK; Eastern Pathology Alliance, Colney Ln, Norwich, UK; Norwich and Norfolk University Hospital, Colney Ln, Norwich, UK; Eastern Pathology Alliance, Colney Ln, Norwich, UK; Norwich and Norfolk University Hospital, Colney Ln, Norwich, UK; Eastern Pathology Alliance, Colney Ln, Norwich, UK; Norwich and Norfolk University Hospital, Colney Ln, Norwich, UK; Eastern Pathology Alliance, Colney Ln, Norwich, UK; Llusern Scientific, Cardiff Edge Business Park, Cardiff, UK

## Abstract

**Background:**

As much as one-third of urine samples which are sent for culture are commonly reported as heavy mixed growth, in line with the current guidelines on UTI reporting. However, it is possible that these may be representative of true polymicrobial infection especially in symptomatic at-risk patient populations. The Lodestar DX UTI is a novel LAMP-based molecular test designed for point of care UTI diagnosis, consisting of a panel of seven uropathogens: *Escherichia coli*, *Enterococcus* species, *Staphylococcus aureus*, *Staphylococcus saprophyticus*, *Proteus mirabilis*, *Pseudomonas aeruginosa* and *Klebsiella pneumoniae*. It was hypothesized that microbial DNA identification may be able to accurately identify clinically relevant levels of uropathogens in mixed growth samples and may aid in improving diagnosis.

**Objectives:**

To determine whether Lodestar DX could be used to accurately detect clinically relevant uropathogens from urine cultures designated heavy mixed growth.

**Methods:**

This work was part of a larger performance evaluation comparing Lodestar DX with standard laboratory culture (flow cytometry and culture). Samples reported as heavy mixed growth by culture had the initial inoculum culture plate reviewed by the evaluation team, to assess whether the Lodestar DX test had accurately identified *visible* relevant uropathogens within the polymicrobial growth.

**Results:**

Of the 241 samples selected for analysis, 35 were reported as heavy mixed growth. Of these, 23 had the inoculum culture plate successfully reviewed and the remaining 12 could not be reviewed; in most cases this was due to the plates being discarded prior to review. Lodestar DX correctly identified at least one relevant uropathogen visible within the growth in 20 of the 23 reviewed culture plate samples. Of the remaining three samples, one contained *E. coli* which was not detected by Lodestar, one contained an unknown coliform not detected by Lodestar, and one sample contained no significant uropathogen growth, but Lodestar detected *E. coli*, *Enterococcus/S. aureus* and *Klebsiella*.

**Conclusions:**

From the polymicrobial infections which were able to be reviewed as part of this evaluation, Lodestar DX correctly identified clinically relevant uropathogens in 87% of the mixed-growth cultures. Whether this represents a true infection or heavily contaminated sample is not clear, but in clinical scenarios where the growth is deemed significant, Lodestar DX is able to accurately identify the common uropathogens present. Identification of the organisms in mixed samples may help guide subsequent therapy and facilitate narrow spectrum prescribing.

